# Sequential MR Images and Radiographs of Epiphyseal Osteomyelitis in the Distal Femur of an Infant

**DOI:** 10.1155/2013/672815

**Published:** 2013-09-21

**Authors:** Hitomi Hara, Toshihiro Akisue, Teruya Kawamoto, Masahiro Kurosaka

**Affiliations:** Department of Orthopaedic Surgery, Kobe University Graduate School of Medicine, 7-5-1 Kusunoki-cho, Chuo-ku, Kobe, Hyogo 650-0017, Japan

## Abstract

Magnetic resonance imaging (MRI) plays an important role in the diagnosis of osteomyelitis, especially during the early phase of the disease. The findings of sequential MRIs during the course of treatment in acute osteomyelitis in children have not yet been reported in the literature. We present a case of acute epiphyseal osteomyelitis in the distal femur of an infant. We monitored imaging changes by sequential MRIs and radiographs. MRI was more useful than radiograph for early diagnosis and evaluation of therapeutic response.

## 1. Introduction

Acute hematogenous osteomyelitis in young children occurs mostly in the metaphysis or the diaphysis of a long bone. Acute hematogenous epiphyseal osteomyelitis is very rare with only a few cases having been reported [1–4]. Early diagnosis and appropriate treatment are important for acute epiphyseal osteomyelitis, because delays can allow the condition to spread from the epiphysis into the metaphysis or the knee joint [2, 4]. This may lead to sequelae of growth plate injury or articular cartilage destruction [1]. Magnetic resonance imaging (MRI) plays an important role in the diagnosis of osteomyelitis, especially during the early phase of the disease. MRI is also useful in evaluating soft tissue involvement, discerning both bone and soft tissue abscesses and showing coexisting articular abnormalities such as effusion or synovitis. The findings of sequential MRIs during the course of treatment in acute osteomyelitis in children have not yet been reported in the literature. In the current patient, we were able to evaluate sequential MRIs during the course of treatment for acute epiphyseal osteomyelitis and find the discrepancy among MR images, clinical findings, and plain radiographs. 

## 2. Case Presentation

A 26-month-old boy was referred to our department with a 10-day history of limping gait due to pain in his right leg. Physical examination and radiographs did not show any findings in the right leg. Two days later, he was admitted to our hospital again with pyrexia. Moreover, local heat and motion pain in the right knee were observed. He repeatedly had infections including sepsis and meningitis after birth because of a primary immunodeficiency disease. His mother had a selective immunoglobulin M (IgM) deficiency. Laboratory examinations on admission showed an elevated white blood cells (WBC) count of 18,900/*μ*L with a left shift and a C-reactive protein (CRP) value of 5.75 *μ*g/mL. Evaluation of immunoglobulin revealed significantly lower serum IgM level (14 mg/dL) and IgG level (657 mg/dL), along with normal IgA level. 

**Figure 1:**
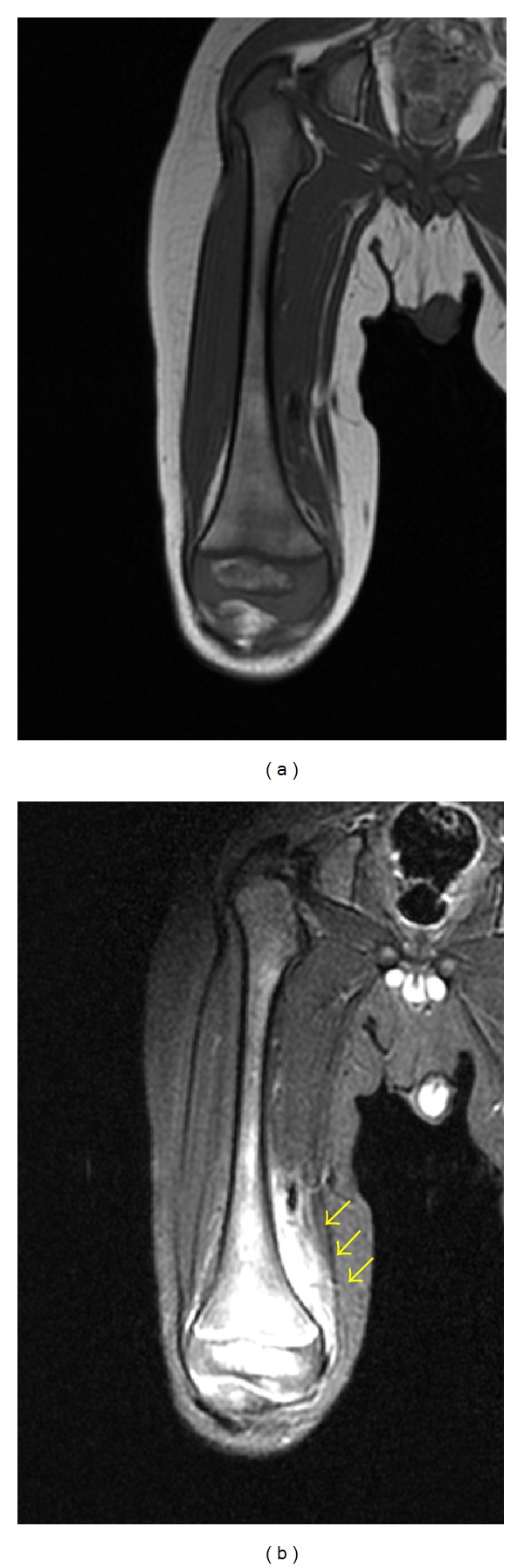
(a) Coronal T1-weighted image of the right femur shows hypointensity in the bone marrow of femoral diaphysis and epiphysis. (b) Coronal STIR sequence shows hyperintensity in the bone marrow of the femoral diaphysis and epiphysis, including the area around the soft tissue.

**Figure 2:**
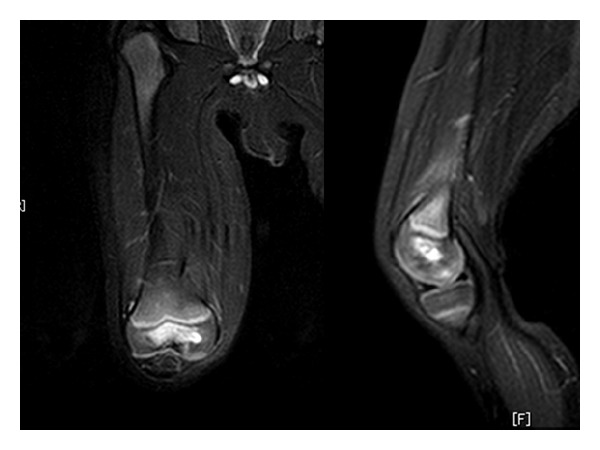
STIR sequences on MRI two weeks after treatment show that bone marrow abnormality and soft tissue edema of the femur have improved. Note the high intensity area in the epiphysis, especially in the medial epiphyseal nucleus.

He was sedated orally with triclofos (70 mg/kg body weight), and MRI was taken. The femoral diaphysis and epiphysis in the bone marrow include hypointensity on T1-weighted images (Figure 1(a)) and hyperintensity on short tau inversion recovery (STIR) sequences (Figure 1(b)). STIR sequences showed high signal intensity in the soft tissue around femur, which suggested extensive infection or reactive change. There was no evidence of joint effusion or abscess. We diagnosed primary acute osteomyelitis of the femur. Blood culture upon admission showed no organism growth. Antibiotic treatment was performed with intravenous cefazolin (1200 mg daily) for two weeks. He became afebrile two day later, and local heat and motion pain in the right knee improved with time. Two weeks after the intravenous antibiotics treatment, CRP was negative and MRI (Figure 2) showed improvement of abnormal intensity in the bone marrow of the femur and the adjacent soft tissue. The femoral epiphysis, especially epiphyseal nucleus, still included hyperintensity on STIR sequences. The MRI suggested a focus of osteomyelitis within the distal femoral epiphysis, just medial of the center, so we diagnosed this lesion as a primary acute epiphyseal osteomyelitis. He was discharged with free gait after two weeks of admission. After the intravenous antibiotics treatment, treatment was changed to oral cefdinir (150 mg daily) for ten weeks. After discharge, recovery was uneventful, and full range of motion without weight bearing was achieved.

**Figure 3:**
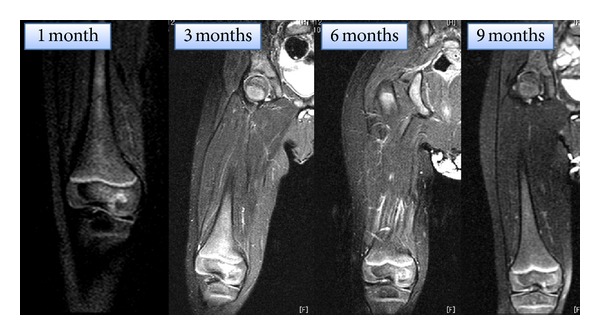
Coronal STIR sequences on repeat MRIs show hyperintensity in epiphyseal nucleus until 6 months later. At 9 months, the intensity returns to normal.

**Figure 4:**
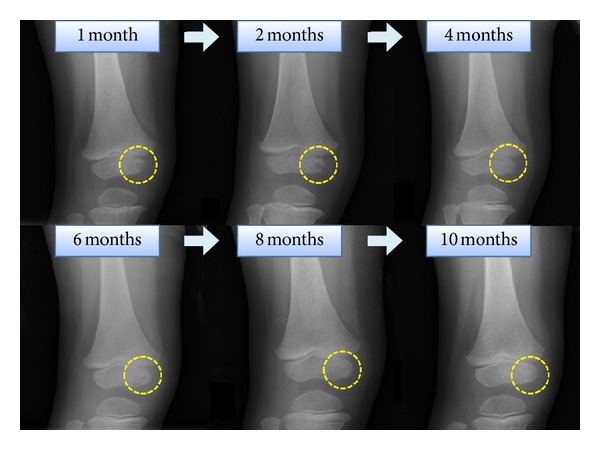
Radiograph at 1 month after onset of disease shows the radiolucency in the medial epiphyseal nucleus. Radiograph at 4 months shows the start of remodeling the epiphysis. Radiograph at 10 months returns almost normal findings.

We investigated sequential radiographs and MRIs throughout the one-year followup. The abnormalities on MRI (Figure 3) improved with the temporal course. The abnormalities as hyperintensity on STIR sequence in epiphyseal nucleus persisted until 6 months after treatment. At 9 months after treatment, the MRI showed normal intensity in epiphyseal nucleus. Epiphyseal cartilage or growth plate destruction was not observed on follow-up MRIs. Sequential radiographs (Figure 4) showed osteolytic lesions in the medial epiphyseal nucleus of the right distal femur at one month after treatment. Radiolucent findings were shown more clearly at two months after treatment. The radiographs showed that remodeling of the epiphyseal nucleus started at four months and that remodeling completed at 10 months after treatment. There was no recurrence of the lesion at followup 2 years after treatment. 

## 3. Discussion

Acute hematogenous osteomyelitis in young children occurs principally in the diaphysis and the metaphysis of the long bone. Acute hematogenous infection of the epiphysis is extremely rare. Kao et al. reviewed 185 cases of acute septic arthritis and hematogenous osteomyelitis in children during 12 years of clinical experience and found only 3 cases of epiphyseal osteomyelitis [1]. We could find only 9 cases of acute epiphyseal osteomyelitis in children that have been reported in English literature. We reviewed 10 cases of acute epiphyseal osteomyelitis in children including our experimental case, and these sites of involvement were distal femur (8 cases), proximal tibia (one case), and proximal radius (one case), and then it occurs predominantly in males (8 cases) than in females (2 cases) [1–3, 5–7]. Epiphyseal osteomyelitis is thought to occur only in infants under 1 year of age, because the vascular channels crossing the growth plate are commonly present in the fetus and infant but disappear by 15 to 18 months of age [8]. Their transphyseal vascular involution with advancing age induces the physis to provide a mechanical barrier to protect against the spread of infection. Trueta and Morgan's detailed report, elucidating the vascular architecture of the epiphysis, provides an explanation for epiphyseal infection [9].

The clinical presentation of acute hematogenous osteomyelitis appears to be changing. Goergens et al. reviewed a large series of children with acute hematogenous osteomyelitis from 1998 to 2002 and compared this data with that of a previous study between 1968 and 1972 [10]. Many patients present with a less florid illness than the classical presentation of a sick child with a high fever and an elevated white blood cells count previously documented. They reported that, although most patients (more than 90%) in their series presented with pain and a limp, an elevated temperature was present in 66% and clinical findings such as local heat, swelling, erythema, or limited adjacent joint motion were present in only 50% of the children. Thirty-two percent of the patients with osteomyelitis had a temperature of less than 37.5°C. Thus, a normal temperature, white blood cells count, or ESR does not exclude the diagnosis of acute osteomyelitis. 

Early diagnosis and appropriate treatment are important for acute epiphyseal osteomyelitis, because delays in the diagnosis and treatment may cause the spread from the epiphysis into the metaphysis or the knee joint. This, in turn, may lead to sequelae of growth plate injury or articular cartilage destruction [1, 2, 4]. The diagnosis of acute hematogenous osteomyelitis is based on clinical findings, supplemented by laboratory examinations and imaging studies. MRI plays an important role in the diagnosis of acute hematogenous osteomyelitis, as pathologic changes on plain radiographs are not evident in the early stages of disease. MRI is both highly sensitive and specific, with reported values ranging from 88% to 100% sensitivity and 75% to 100% specificity, respectively [4, 11]. MRI is useful in defining the degree of soft tissue involvement, identifying both bone and soft tissue abscesses, and showing coexisting articular pathologies such as effusion or synovitis. In the current patient, T1-/T2-weighted and STIR sequences on initial MRI showed a large effusion and marrow edema of the femur and the surrounding soft tissue, and signal intensity changes at both epiphysis and diaphysis of the femur were observed. There was no discerning abscess or joint effusion. We diagnosed this case as acute hematogenous osteomyelitis of the femur from clinical and MRI findings and started treatment by intravenous antibiotics. The MRI after 2 weeks confirmed successful treatment and provided additional findings to prove that the origin of the infection was the epiphyseal nucleus. These findings suggest that sequential MRIs may be useful not only for diagnosing acute hematogenous osteomyelitis in the early stages but also for monitoring treatment response and clarifying the localization of the disease. We followed this patient radiologically by sequential MRIs and plain radiographs because he had frequent previous infectious diseases due to immune deficiency. According to past reports [2, 3, 5], initial radiographs usually show negative findings, and sequential radiographs (at 10 to 21 days later) show lytic lesions of the epiphysis. Furthermore, the radiographic changes detected in acute hematogenous osteomyelitis may only be about 20% at 10 to 14 days after the onset of illness. In the current patient, a radiograph at one month after treatment showed osteolytic change in the medial epiphyseal nucleus of the right distal femur, and radiographs revealed the start of remodeling of the epiphysis four months after treatment. Radiographs were more useful to evaluate the remodeling process of the epiphysis than MRI. To our knowledge, this is the first report showing sequential MRIs in acute epiphyseal osteomyelitis in children. STIR sequences on sequential MRI at 1, 3, and 6 months showed the high intensity area of the epiphysis although the clinical findings revealed no evidence of recurrent osteomyelitis. We need to consider that signal change in MRI may persist for 6 months when we evaluate musculoskeletal MRIs in children after epiphyseal osteomyelitis.
